# Accelerated Predictive Stability Testing: Accelerating Registration Phase and Application of Reduced Designs for Shelf-Life Determination of Parenteral Drug Product

**DOI:** 10.3390/pharmaceutics17020160

**Published:** 2025-01-25

**Authors:** Lara Pavčnik, Mateja Prunk, Tina Trdan Lušin, Robert Roškar

**Affiliations:** 1 Sandoz Development Center Slovenia, Lek Pharmaceuticals d.d., Verovškova 57, SI-1526 Ljubljana, Slovenia; lara.pavcnik@sandoz.com (L.P.); mateja.prunk@sandoz.com (M.P.); tina.trdan_lusin@sandoz.com (T.T.L.); 2 Department of Biopharmaceutics and Pharmacokinetics, Faculty of Pharmacy, University of Ljubljana, SI-1000 Ljubljana, Slovenia

**Keywords:** shelf life, accelerated stability assessment program, stability study, degradation kinetics, parenteral dosage forms

## Abstract

Objectives: This article explores the applicability of the accelerated stability assessment program (ASAP) in stability studies for parenteral medications. Conventional stability testing requires extensive evaluation over the entire shelf life of a product, which can be very time-consuming. In contrast, ASAP provides an efficient approach to support drug product development and expedite regulatory procedures. Methods: The study involved subjecting the medication to different stress and long-term stability conditions and monitoring the formation of degradation products. A systematic methodology was employed to evaluate the stress stability data of the parenteral medication using various designs (full and reduced). ASAP models were then developed from these data and assessed using the statistical parameters R^2^ (coefficient of determination) and Q^2^ (predictive relevance). To validate the accuracy of the models, the predicted levels of degradation products from each of the 13 models were compared with the actual long-term stability results using the relative difference parameter. Results: The results confirmed the suitability of the evaluated full model and 11 reduced models for predicting degradation products, except for the two-temperature model, demonstrating the effectiveness of ASAP in stability studies and providing reliable predictions. However, the three-temperature model was identified as the most appropriate model for the parenteral medication under investigation. The statistical analyses showed high R^2^ and Q^2^ values, indicating robust model performance and predictive accuracy. Consequently, we applied the selected model on various formulations, demonstrating the suitability of the model and impurity levels below the ICH specification limit. Conclusions: This research enhances understanding of how ASAP designs can be applied to stability studies for parenteral medications and demonstrates the significance of the application of ASAP during drug product development to expedite the initiation of procedures and implement post-approval variations.

## 1. Introduction

The purpose of stability testing is to provide evidence on how the quality of a drug product varies with time under the influence of a variety of environmental factors, such as temperature, humidity, and light. In addition, all dosage forms must be stable under pre-determined manufacturing, packaging, and usage conditions. The aim of the stability study is, therefore, to assess the changes during the study and to establish the shelf life and recommended storage conditions for the drug product. The traditional design, in line with ICH Q1A (R2), is to perform long-term stability testing every three months in the first year, every six months in the second year, and once a year throughout the proposed shelf life. Long-term conditions should correspond to the storage conditions indicated on the container label (e.g., refrigerator at 2–8 °C or 25 ± 2 °C/60% RH ± 5% RH or 30 ± 2 °C/65% RH ± 5% RH). Drug products should also be stored under accelerated conditions, which is at 40 ± 2 °C/75% RH ± 5% RH or at 25 ± 2 °C/60% RH ± 5% RH, for six months, tested at least at zero, three, and six months in the case of drug products with storage conditions at room temperature and refrigerator, respectively. Furthermore, when long-term studies are conducted at 25 ± 2 °C/60% RH ± 5% RH and significant change occurs at any time point during six months of testing under accelerated conditions, additional testing at an intermediate storage condition should be performed. Additionally, stress stability studies are conducted to evaluate the effect of short-term excursions outside the label storage conditions (such as might occur during transport). As per guidelines, the drug product is subjected to elevated temperatures for a specific duration, usually one month. For submission, the long-term testing should cover a minimum of 12 months’ duration on at least three primary batches. Furthermore, to confirm the storage conditions and proposed shelf life, real-time long-term data have to be available [1].

The ICH approach primarily aims to confirm the stability of a product rather than predict it. Therefore, it is essential that the entire proposed shelf life is covered by long-term stability testing. Although limited extrapolation is allowed during the submission process under specific conditions, such as when long-term and accelerated data demonstrate minimal or no change over time, the proposed shelf life can be up to twice, but not more than 12 months beyond, the period covered by long-term data [2]. However, it is worth noting that despite the statement in ICH Q1A (R2) that “alternative approaches can be used when there are scientifically justifiable reasons” for marketing applications, the exploration of alternative stability approaches has been limited. This is because there is a lack of comprehensive guidance regarding what constitutes a regulatory acceptable alternative stability approach. As a result, the pharmaceutical industry and regulators have yet to fully embrace and implement alternative approaches for marketing registration [3].

The IQ Consortium Science- and Risk-based Stability Working Group was formed in 2015 to conduct a comprehensive review of ICH Q1A(R2) and its annexes [4] The group identified that the current guidelines do not fully incorporate modern tools and strategies, such as stability risk assessment (SRA) or risk-based predictive stability (RBPS) tools, in the design of stability programs and compilation of stability data packages for registration applications. Additionally, stress studies conducted during the registration stability study often have limited data, as only one elevated temperature is typically used for a month. This single stress condition may not capture all potential degradation pathways and conditions that could affect product stability throughout its shelf life. These deficiencies in instructions and limited data can result in delays in the registration process, leading to delayed access to critical medications for patients [5,6].

To obtain a more comprehensive understanding of the impact of various stress conditions throughout the shelf life of the product, additional stress studies beyond the scope of the registration stability studies that are prescribed in the current guidelines can be conducted. These supplementary stress studies can include different higher temperatures and time points. By expanding the stress studies to include a wider range of conditions and time points, a more thorough evaluation of the product’s stability profile and potential degradation pathways can be achieved.

Kinetic studies of chemical degradation in solid pharmaceutical materials have been a topic of extensive research. Several models and methodologies have been reported in academic research institutions, but these approaches have rarely been applied in routine pharmaceutical company drug development programs [7,8,9,10]. This is mainly due to the limitations of the methodologies for pharmaceutical solids and the expertise required to conduct such studies. In 2007, Waterman and colleagues introduced an accelerated stability assessment program (ASAP) for stability modeling of pharmaceutical materials [11]. ASAP was developed based on the moisture-modified Arrhenius equation and the isoconversional model-free approach. It provided a practical protocol for routine stability testing in a regular pharmaceutical analysis laboratory. The successful applications of ASAP and the commercial availability of ASAPprime, a software specifically designed for supporting ASAP studies, have contributed to evaluate accelerated predictive stability studies in numerous scientific articles.

In the past, ASAP studies were applied for drug stability testing with different APIs [12,13,14], changes in excipients [15], changes in packaging configuration [16], and process parameters [17]. Most of the mentioned studies were performed on solid dosage forms in early-stage development; however, none were used for registration purposes [11,14,18,19]. Limited data regarding accelerated predictive stability (APS) studies are available for parenteral pharmaceutical dosage forms. Furthermore, the reported studies do not involve evaluating various temperature models and identifying the most appropriate reduced model to predict the drug’s behavior over its shelf life.

Therefore, the aim of this study was to (1) establish a basic ASAP model for a carfilzomib parenteral drug product to support drug product development and early initiation of regulatory procedures; (2) optimize the full ASAP model while maintaining predictive reliability to further accelerate stability evaluation; (3) demonstrate the applicability of the ASAP model for different post-approval changes (e.g., changes in the manufacturing process, formulation,…) to speed up the submission of regulatory variations; and (4) confirm the reliability of the developed ASAP models by correlating predictions and real-time stability data and consequently encourage researchers and authorities to confidently utilize accelerated stability data as a reliable indicator of long-term stability.

## 2. Materials and Methods

### 2.1. Drug Product

Sandoz has developed a parenteral pharmaceutical product called carfilzomib, which is administered through intravenous infusion, and 1 mL of the product contains 10 mg of carfilzomib. It is produced in a filling volume of 60 mg/6 mL and packed in a clear, colorless type I glass vial with a bromo butyl rubber stopper and aluminum crimp cap.

### 2.2. Stability Study Design

A single laboratory development batch (prototype) was manufactured and stored in stability chambers to evaluate its stability under various conditions. To predict the behavior during shelf life, a long-term stability study at a controlled temperature of 5 ± 3 °C, tested at 0, 3, 6, 12, and 24 months, and an accelerated stability study at 25 ± 2 °C/60% RH ± 5% RH, tested at 1, 3 and 6 months, were performed. In addition, samples were subjected to a stress study, where higher temperatures and humidities were used. These included conditions at 30 ± 2 °C/65% RH ± 5% RH for 1 month, tested at 14 days and 1 month; at 40 ± 2 °C/75% RH ± 5% RH for 21 days, tested at 7 and 21 days; at 50 ± 2 °C/75% RH ± 5% RH for 14 days, tested at 7 and 14 days; and at 60 ± 2 °C/75% RH ± 5% RH for 7 days, tested at 1 and 7 days. Throughout these stability studies, the vials were placed in an upright orientation.

The stability study design also involved three additional laboratory formulations that differed from the prototype in terms of the acid used for pH correction. These formulations also varied from each other in terms of the final pH of the product. All three formulations were subjected to stability conditions at 40 ± 2 °C/75% RH ± 5% RH, tested at 7 and 21 days, at 50 ± 2 °C/75% RH ± 5% RH, tested at 7 and 14 days; and at 60 ± 2 °C/75% RH ± 5% RH, tested at 1 and 7 days.

### 2.3. Tested Parameters and Analytical Methods

**Figure 1:**
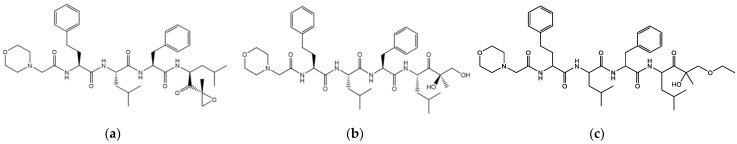
Structure of the examined compound and monitored degradation products: (**a**) carfilzomib, (**b**) diol, and (**c**) ethyl ether.

During the stability studies, testing of the following degradation products was performed: diol impurity, ethyl ether impurity, and total impurities. The mentioned identified impurities are ones that increased significantly during stability studies and reached levels that were above qualification limits. Consequently, predictive stability data were needed to properly set the protocol for the impurity qualification study. Structures of the examined compound (carfilzomib) and monitored degradation products are presented in Figure 1.

A validated ultra-high performance liquid chromatography (UHPLC) method was used to determine the degradation products. The chromatographic conditions for the gradient UHPLC method are summarized in Table 1.

The standard solution for determination of impurity levels was prepared in concentrations of 2.5 µg/mL of carfilzomib. Analyzed samples were prepared from liquid to sample solutions with a concentration of 0.5 mg/mL of carfilzomib. The solvent used for the sample and standard solution preparation was ethanol.

### 2.4. Reduced Designs

To evaluate the stability data, several reduced designs were used, involving different combinations of temperature conditions, and they are presented in Table 2.

### 2.5. Data Analysis

Stability data were evaluated with the statistical program ASAPprime^®^, version 6.0.2 (FreeThink Technologies Inc., Branford, CT, USA). Mathematical calculations were based on the use of the modified Arrhenius equation, which describes the relationship between the rate of degradation and temperature/humidity:

(1) $$\mathrm{ln k}=\ln A-\frac{E_{a}}{RT}+B\;(RH)$$

In the above equation, k is the degradation rate constant; A is the preexponential factor related to the probability of the molecules colliding with the correct orientation so that the collision gives rise to the product’s preexponential factor; E_a_ is the activation energy of the reaction, typically measured in kJ mol^−1^ or kcal mol^−1^, which describes the “temperature sensitivity” of the drug; R is the universal gas constant with values of 8.314 J K^−1^ mol^−1^ or 1.987 Cal K^−1^ mol^−1^; T is the absolute temperature expressed in Kelvin degrees; B is a coefficient that represents the “humidity sensitivity” of the drug; and RH is the relative humidity expressed as a percentage (%) [18,20]. Since our product is liquid in a vial, humidity part (B (RH)) of the modified Arrhenius equation was not considered; therefore, the below equation was used:

(2) $$\mathrm{ln k}=\ln A-\frac{E_{a}}{\left(RT\right)}$$

The quality of the resulting model was assessed by R^2^ and Q^2^. R^2^ is an indication of the fit quality of the data to the appropriate equation, while Q^2^ is an indication of the ability of the model to predict results. In practice, an adequate model would give a value of R^2^ no smaller than about 0.85. Q^2^ measures how well the model performs, derived from the leave one out cross validation method. It also has a maximum value of 1 but can be negative for poor models. While R^2^ uses all the data points to fit a single plane, the algorithm used to calculate Q^2^ ignores one data point, fits a plane using the remaining data, and then sees how well ln (k) is predicted for the omitted data point. It then repeats this loop (dropping one out, fitting a plane, and predicting ln (k) for the dropped data point) for each remaining data point. In practice, Q^2^ will be less than R^2^, but the difference should be no more than about 0.2 for an adequate model [13].

For the ASAP evaluation, stability data were used with three decimal places. This level of precision was maintained to ensure accurate calculations. However, predicted values for impurities were reported to two decimal places, as per the ICH Q3B guideline [21]. To assess the comparability between predicted (P) and actual (A) impurity data, we calculated the relative difference with the below equation:

(3) $$\mathrm{Rel}.\mathrm{difference}\;(\%)=\frac{\;IA-PI}{\frac{\left(A+P\right)}{2}}\times 100$$

We also established an acceptable limit for our data, determining that a relative difference of 30% or lower is considered acceptable. This limit was set by considering the criteria’s intermediate precision from the method validation for impurity levels below 0.2%.

## 3. Results

The laboratory development batch (prototype) was tested at all stability conditions described in Section 2.2. Diol impurity and ethyl ether impurity increased the most significantly during the stability studies; therefore, those two impurities were selected as critical quality attributes or as stability-indicating parameters. Additionally, total impurities were also monitored. The results for impurities of the prototype and three different formulations are presented in the Supplementary Materials (Table S1).

### 3.1. ASAP Evaluation of the Full Model (FM)

The results for the prototypes from five different temperature stability conditions (Table 2) were evaluated with the statistical program ASAPprime. The predicted values of impurities (diol and ethyl ether) and total impurities for condition 5 ± 3 °C obtained after statistical evaluation of the full model (FM) are presented in Table 3. Furthermore, in Table 3, the actual stability data at condition 5 ± 3 °C are also presented.

**Figure 2:**
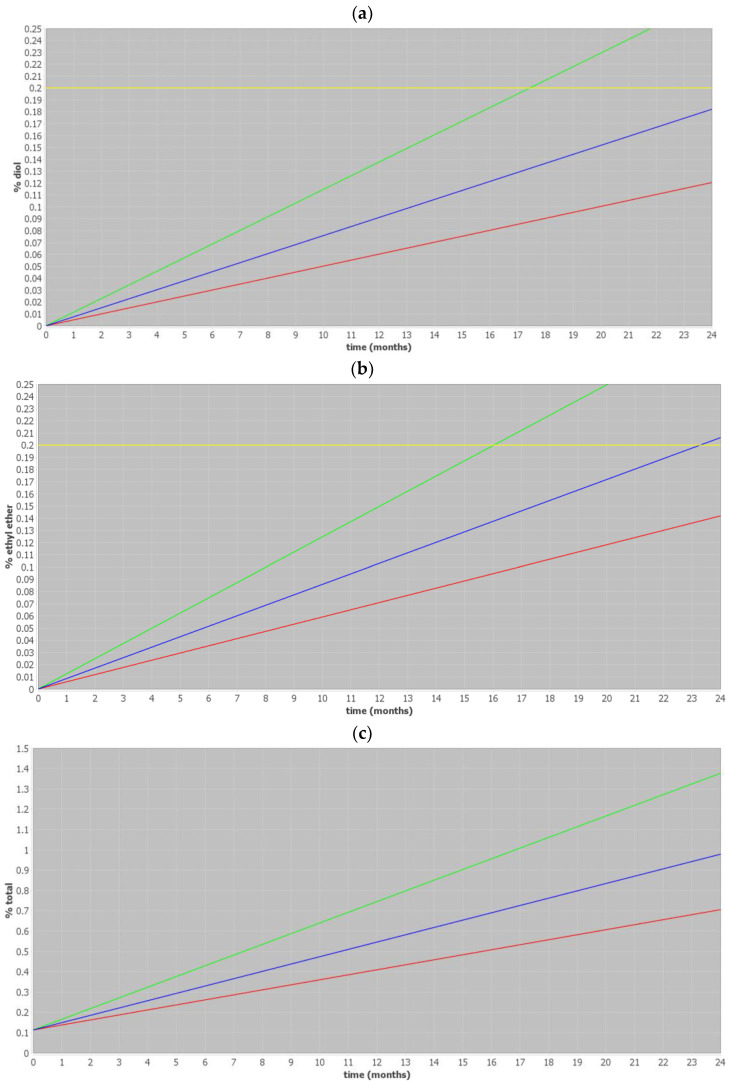
ASAP prediction for (**a**) diol, (**b**) ethyl ether, and (**c**) total impurities, where the blue line represents the average result, the green line represents the upper 95% confidence limit, the red line represents the lower 95% confidence limit, and the yellow line represents the ICH specification limit of 0.2%. An ICH limit is not presented for figure (**c**) since, in line with ICH guidelines, a limit for total impurities is not given.

A graphical presentation of the ASAP prediction is shown in Figure 2. The figures below show the predicted increase in degradation products during the shelf life of the drug product. These degradation products were determined to be critical attributes, since the levels, presented with a blue line, were close to the specified limit of 0.2%. We also show the upper 95% confidence limit as a green line which crosses the 0.2% specification limit; however, its relevance is limited in the context of drug development. This is because the ASAP evaluation process is utilized to determine the most suitable model for predicting impurities and to establish appropriate shelf-life limits for those impurities that either approach or exceed the ICH limit of 0.2% [21]. The upper 95% confidence limit becomes relevant during the registration phase of drug development, as it plays a crucial role in predicting the shelf life of the drug product based on the observed increase in impurities over time [2].

### 3.2. ASAP Evaluation of the Four-Temperature Models

To assess the impact of storage conditions, different four-temperature reduced models (Table 2) were evaluated with the statistical program ASAPprime. The actual results for the condition of 5 ± 3 °C and the predicted values obtained after the statistical evaluation of the reduced models are presented in Table 4. The data of the full model (FM) are also included for comparison. To assess the adequacy of the reduced models, statistical parameters are provided in Table 4. The evaluated models demonstrate a strong fit to the data, as evident from the high values of the R^2^ parameter. Furthermore, the high values of the Q^2^ parameter indicate the accuracy of the predictions made by these models.

### 3.3. ASAP Evaluation of the Three-Temperature Models

The ASAP prediction was conducted using three-temperature models, presented in Table 2. Models M5–M10 were designed considering all combinations of temperatures except for 25 °C, which was used in each model. M11, however, was designed with only extreme temperatures: 60 °C, 50 °C, and 40 °C. The full model and reduced models are compared with predicted impurity values at 6, 12, and 24 months in Table 5. To assess the adequacy of the reduced models, statistical parameters are also provided in Table 5. The evaluated models demonstrate a strong fit to the data, as evident from the high values of the R^2^ parameter. Furthermore, the high values of the Q^2^ parameter indicate the accuracy of the predictions made by these models.

### 3.4. ASAP Evaluation of Accelerated and Stress Conditions

For registration purposes, to increase the rate of chemical degradation or physical change of a drug product, stability testing was performed by using elevated storage conditions as part of the formal stability study. Data from these studies can be used to evaluate the effect of short-term moves outside the labeled storage conditions, such as those that might occur during shipping [1]. Therefore, an ASAP study was performed on data of an accelerated condition at 25 ± 2 °C/60% RH ± 5% RH (time points initial, 3 m, and 6 m) and one stress condition at 40 ± 2 °C/75% RH ± 5% RH (time points 7 and 21 days). The predicted values for conditions at 5 ± 3 °C obtained after ASAP evaluation of model M12 are presented in Table 6. The table below also displays statistical parameters R^2^ and Q^2^. However, their relevance is limited in this context since a linear fit was only implemented between two points.

### 3.5. ASAP Evaluation of Different Formulations

**Figure 3:**
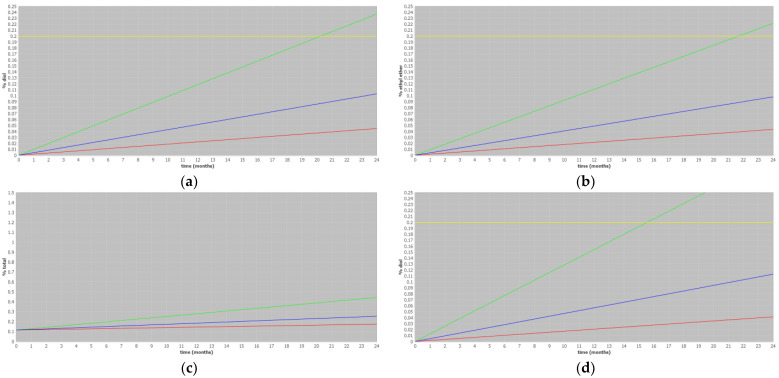

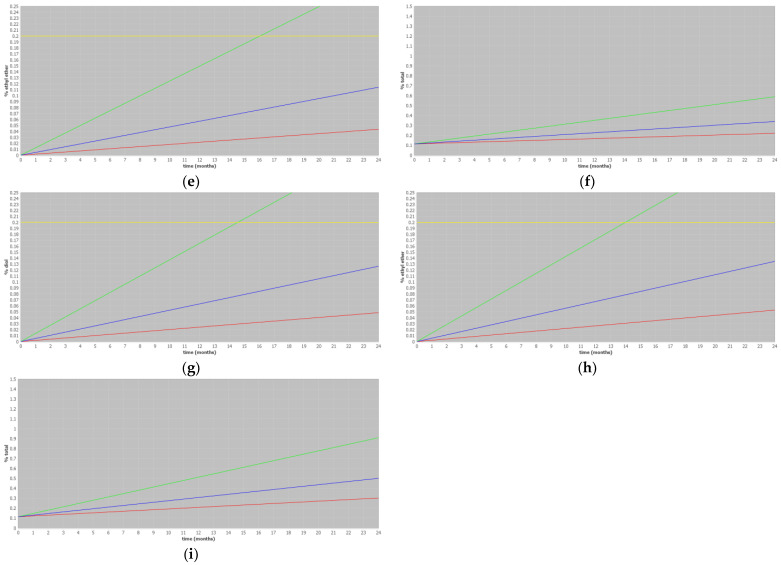
ASAP prediction for (**a**) diol impurities of formulation F1, (**b**) ethyl ether impurities of formulation F1, (**c**) total impurities of formulation F1, (**d**) diol impurities of formulation F2, (**e**) ethyl ether impurities of formulation F2, (**f**) total impurities of formulation F2, (**g**) diol impurities of formulation F3, (**h**) ethyl ether impurities of formulation F3, and (**i**) total impurities of formulation F3, where the blue line represents the average result, the green line represents the upper 95% confidence limit, the red line represents the lower 95% confidence limit, and the yellow line represents the ICH specification limit of 0.2%. An ICH limit is not presented in figures (**c**,**f,i**) since, in line with ICH guidelines, a limit for total impurities is not given.

The ASAP prediction was conducted for three distinct formulations of carfilzomib which differed from the prototype and were tested at a later stage in the development process, focusing on model M11. The statistical parameters obtained after ASAP evaluation are presented in Table 7. The evaluated models demonstrate a strong fit to the data, as evident from the high values of the R^2^ parameter. Furthermore, the high values of the Q^2^ parameter indicate the accuracy of the predictions made by these models. Furthermore, a graphical presentation of ASAP prediction is shown in Figure 3. The figures below show the predicted increase in degradation products during the shelf life of different formulations. The blue line indicates the predicted increase in impurity levels, which remains below the specified limit of 0.2% for all degradation products, diol impurities, and ethyl ether impurities.

### 3.6. Evaluation of Comparability Between Different Temperature Models

**Figure 4:**
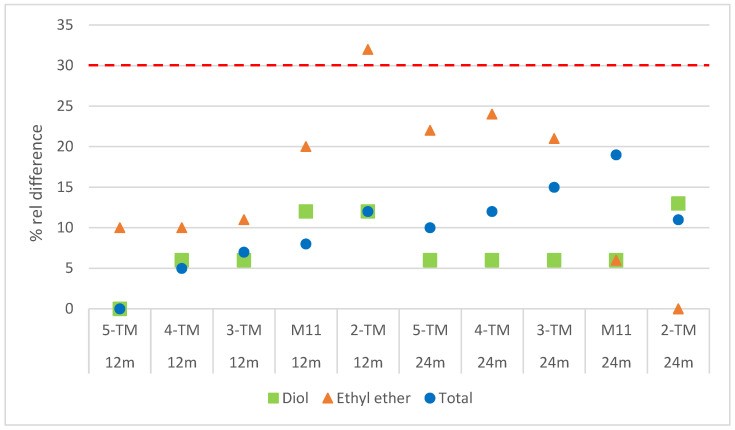
Comparability between different temperature models with the parameter of relative difference, where 5-TM represents the 5-temperature model (FM), 4-TM represents the average of 4-temperature models (M1–M4), 3-TM represents the average of 3-temperature models (M5–M10), and 2-TM represents the 2-temperature model (M12). The red line represents the relative difference limit of 30%.

Comparisons were made between different temperature models (5-TM, 4-TM, 3-TM, and 2-TM) obtained from Table 4, Table 5 and Table 6 using the parameter of relative difference. The relative difference between the actual and predicted data for each impurity was calculated and is presented in the Supplementary Materials (Table S2). Average relative difference values were calculated for four temperature models (M1–M4) and three temperature models (M5–M10). M11 was treated separately from the other three temperature models as it represents extreme temperatures. The graphical representation of these results can be observed in Figure 4.

Figure 4 shows the calculated relative differences for the 12-month and 24-month time points. The exclusion of the 6-month time point from the evaluation was due to the low levels of impurities observed (below 0.05%). Consequently, those levels of impurities below the reporting threshold may contribute to higher values of the relative difference, primarily due to the variability of the analytical method. The fact that only one measurement is performed at a given time point contributes to this variability, as it can be influenced by various factors at very low levels, including random errors or occasional anomalies that can lead to values outside the expected range.

## 4. Discussion

Our study aimed to achieve several key objectives in the context of developing parenteral dosage forms. Firstly, we sought to establish a basic ASAP (accelerated stability assessment program) model to support drug product development and to accelerate the initiation of regulatory procedures. Secondly, we focused on optimizing the ASAP model while maintaining its predictive reliability and consequently further decreasing the time needed for stability evaluation. Thirdly, we aimed to demonstrate the applicability of the developed ASAP model across various drug product compositions for faster initiation of regulatory variations. Finally, we intended to confirm the reliability of the developed ASAP models by correlating their predictions with real-time stability data.

### 4.1. Stability Study Design

The study started with a review of the available literature on carfilzomib stability and an experimental evaluation of carfilzomib in liquid form in a vial to determine critical quality attributes for stability evaluation and prediction. It turned out that diol and ethyl ether impurities would dictate the shelf life and storage conditions of the tested drug product, since the ASAP prediction showed that impurity levels either approach or exceed the ICH limit of 0.2%, as shown in Figure 2. The carfilzomib impurity profile has already been effectively studied by Sestak et al. [22], where it was found that the diol impurity is formed from carfilzomib via acid hydrolysis of epoxide ring. Namely, the target pH of the formulation is 3.0–3.1, and although no water is intentionally added to the formulation, there is some residual water from the drug substance, excipients, and environmental conditions that is sufficient in order for hydrolysis to occur. On the other hand, ethyl ether impurity is formed as a consequence of ethanol addition to the epoxy group of carfilzomib. Namely, absolute ethanol is used as a main excipient/solvent in the formulation.

As parenteral drug products are typically packed in non-permeable containers such as glass vials, as is the case of the carfilzomib drug product, the impact of humidity on the stability can be considered negligible. Therefore, the main focus of this stability study is the effect of temperature. However, it is important to note that humidity is still listed in the storage conditions as stability chambers are set to the specified temperature and humidity conditions. Given the approach to focus primarily on temperature as the key factor affecting stability, the Arrhenius equation used in ASAP was simplified to effectively assess the degradation of the drug product over time at different temperature points.

Based on development data, the proposed long-term storage condition for the carfilzomib drug product is in a refrigerator at a temperature of 5 ± 3 °C. To assess the proposed specification limits and determine the appropriate shelf life for the product, an extensive stability study was conducted. This study involved subjecting the product to various higher temperature conditions using a full stress model consisting of five temperatures. By evaluating the degradation products under full and different reduced temperature models and monitoring their growth over time, the study aimed to determine the optimal specification limits and appropriate storage conditions and establish a reliable shelf life for the carfilzomib drug product.

In the past, ASAP studies have mostly focused on solid dosage forms, leaving limited data available for parenteral pharmaceutical dosage forms. Consequently, our study represents a significant contribution as the first attempt to evaluate different temperature models and identify the most suitable reduced model for predicting the behavior of the drug over its intended shelf life in parenteral dosage form.

### 4.2. Comparison Between Full and Reduced Models

In accordance with ICH Q1A (R2) guidelines, stability studies for registration purposes primarily involve testing the drug product at a few elevated temperatures, including accelerated conditions and intermediate conditions, where applicable [1]. The purpose of utilizing these elevated storage conditions is to accelerate the rate of chemical degradation in the drug product and consequently facilitate the assessment of its stability. However, it is important to note that these limited stress conditions may not fully encompass all potential degradation pathways and conditions that could impact product stability throughout its intended shelf life. To address this gap, we conducted a comprehensive stress study involving five temperatures, which is, for our study, a full temperature model. We further evaluated the comparability of the results obtained with different reduced temperature models.

During the primary analysis, the data from the full model, including five stability conditions, was evaluated using ASAP. In the subsequent step, the data were analyzed using reduced models, the four-temperature, three-temperature, and two-temperature models. This allowed for the prediction of impurities under long-term conditions and the determination of statistical parameters for each model.

The statistical parameters presented in Table 4 and Table 5 demonstrate that both the four-temperature (M1–M4) and two-temperature (M5–M10) reduced models show a strong fit to the data, as indicated by the R^2^ parameter. This indicates that these models effectively capture the relationship between variables and explain a substantial portion of the data’s variability. The accuracy of the predictions made by these models is further emphasized by the Q^2^ parameter, validating their reliability and effectiveness in analyzing and interpreting the data. However, when examining the statistical parameters obtained with the two-temperature model (M12) in Table 6, it is apparent that there are no results presented for the Q^2^ parameter. This is because the ASAP prediction was performed using only two temperatures. The limited amount of data also led to unreliable results for parameter R^2^, which evaluates the goodness of fit for a model. With such a small dataset, it is not adequate to accurately determine the relationship between variables, resulting in a high R^2^ value, such as 1.

The predicted values for each model, both full and reduced models, were assessed using the parameter of relative difference. This parameter was calculated between the predicted and actual results for the 12-month and 24-month time points and is shown in Figure 4. Most of the calculated relative differences fall within the acceptable limit of 30%. However, there is one result from the two-temperature model that exceeds this acceptable limit. These observations underscore the significance of recognizing the limitations of the two-temperature model and the necessity of incorporating additional data points or conditions to enhance the reliability and accuracy of the predictions. Including additional temperature conditions would provide a more comprehensive understanding of the degradation pathways and stability profile of the drug product, leading to more accurate predictions.

In summary, after assessing the statistical parameters and relative difference values, it can be concluded that the reduced models, the four-temperature (M1–M4) and three-temperature (M5–M10) models, exhibit comparable performance and predictive capability to the full model. This indicates that these reduced models are reliable and effective in evaluating the stability and predicting impurity values of the drug product.

By using one of the reduced models (M1–M10), it would take 3 months to evaluate the stability of a parenteral drug. Therefore, to evaluate if an accurate prediction of impurities can be assessed in shorter time (21 days), an additional three-temperature model (M11) was evaluated, where only three extreme temperatures—60 °C, 50 °C, and 40 °C—were considered. The subsequent ASAP evaluation demonstrated that the statistical parameters were comparable to those of the full model, as shown in Table 5. Furthermore, the calculated relative differences fell within the acceptable limit of 30%.

Traditionally, stability testing involves using one elevated storage condition along with an accelerated stability condition as part of the formal stability study. As a result, an ASAP evaluation can currently be performed with a two-temperature model. However, by incorporating a higher number of temperatures in the model, the complex relationships between temperature and impurities can be better captured, resulting in improved predictive capabilities, which allows for a more robust assessment of the drug product’s stability over its intended shelf life. For the carfilzomib drug product, which is packed into an impermeable container closure system, our study demonstrated that even the three-temperature model (M11) was sufficient to adequately predict levels of diol, ethyl ester, and total impurities within a shorter timeframe.

### 4.3. Application of the Three-Temperature Model for Different Formulations

During the product’s life cycle, various post-approval changes can occur after the initial registration of a pharmaceutical product. These changes can involve different aspects such as the active pharmaceutical ingredient (API), the formulation, process parameters, and other related factors. These post-approval changes are typically made to optimize the product, improve its quality, enhance manufacturing efficiency, or address regulatory requirements. Therefore, assessing the suitability of post-approval batches as soon as possible is crucial to ensure product quality and compliance. The application of ASAP can be highly beneficial in this regard to evaluate the stability of the new batches in a shorter period compared to traditional stability testing.

In Section 4.2, we evaluated different reduced models. The statistical parameters and calculated relative differences between the actual and predicted values show that all three-and four-temperature models are comparable to the full model. Thus, the shortest reduced model (M11) can be used to predict impurity behavior during shelf life. To assess the suitability of the extreme three-temperature model for other carfilzomib parenteral formulations, data from three different development batches were evaluated using ASAP. It is important to note that these formulations differ from the prototype in terms of the acid used for pH adjustment and the final pH of the product.

The results of the ASAP models for all three formulations (Table 7) demonstrate a strong fit to the data, as evidenced by the statistical parameters R^2^ and Q^2^. This indicates that all the models accurately predicted the impurity levels throughout the product’s shelf life. Figure 4 visualizes the ASAP predictions for diol and ethyl ether impurities, confirming that the increase in impurities remains below the ICH limit of 0.2% even after 24 months of storage. This suggests that the changes in pH, which could be considered as post-approval changes, did not have a significant impact on the impurity levels and remained within acceptable limits.

Comparing our study’s results with previous ASAP studies conducted for drug stability testing with different APIs [12,13], changes in excipients [15], and changes in packaging configurations [16], we can identify similarities in the conclusions regarding the applicability of ASAP studies. However, it is important to note that compared to the listed studies, performed on solid dosage forms, our study was performed on parenteral drug products. In previous studies on APIs, it was determined that a retest period could be established using ASAP studies. This finding is comparable to our study, as one of our objectives was to confirm the product’s shelf life using the ASAP model. Additionally, Natalie et al. utilized ASAP studies to investigate the impact of different excipients on the physical aging of tablets, which is similar to our study where we investigated the effect of pH in different formulations. Furthermore, Viktoria et al. employed ASAP studies to predict the presence of degradants during the shelf life of a drug product packed in various packaging configurations. Their results demonstrated minimal differences between the predicted and actual results. This finding aligns with our study, although we compared the results using the relative difference parameter. These similarities between the results of the different studies support the utility and effectiveness of ASAP studies in different areas of drug development and stability assessment. Furthermore, the use of the ASAP model for stability studies during the registration phase enables the implementation of reduced matrixing designs, as studied by Pavčnik et al. [23]. This approach enables a more efficient use of resources by reducing the number of stability samples tested while providing reliable and accurate predictions of product stability.

## 5. Conclusions

In the development of generic drug products, it is critical that development progresses rapidly and that the generic drug product is available on the market quickly to meet patient needs. While numerous studies have demonstrated the effectiveness of ASAP in the early stages of development to select optimal prototype formulations, our research uniquely focuses on the regulatory aspects associated with parenteral drug products in impermeable container closure systems. In this article, we present an ASAP study for a parenteral drug product investigating the influence of temperature on degradation. Our results show that reduced models have comparable performance and predictive ability compared to the full model. Thus, all reduced models, except for the two-temperature model, can be used effectively in stability design. Among the reduced models, M11 proves to be particularly suitable in terms of time points and study duration.

In summary, our study confirms that ASAP modeling could be very important as a supportive stability package for dossier submission, as it improves understanding of the stability of the developed drug product and consequently could enable faster initiation of approval procedures. In addition, it could enable quick assessment, implementation, and approval of minor changes in the post-authorization phase. By achieving these goals, we aimed to encourage researchers and regulators to confidently utilize accelerated stability data as a reliable indicator of long-term stability. This, in turn, can speed up the drug development process and lead to faster access to safe and effective medicines for patients. Based on the presented study, we believe that the presented approach could be applied as an alternative approach for stability extrapolation.

## Figures and Tables

**Table 1:** Chromatographic conditions of UHPLC method for determination of degradation products.

Degradation Products			
Mobile phase A	1.25 mM buffer NH_4_H_2_PO_4_ (pH = 6.0): Acetonitrile = 70:30 (*v*/*v*)		
Mobile phase B	Acetonitrile: Purified water = 90:10 (*v*/*v*)		
Column	Acquity BEH C18, 1.7 µm, 150 × 2.1 mm		
Column temperature	45 °C		
Flow rate	0.4 mL/min		
Detection wavelength	210 nm		
Gradient parameters	Time (min)	%A	%B
	0	100	0
	1	100	0
	35	60	40
	43	20	80
	45	20	80
	45.1	0	100
	65	0	100
	65.1	100	0
	72	100	0

**Table 2:** List of the reduced designs.

Design	Temperature/Time Points										No. of Temperatures
	25 °C		30 °C		40 °C		50 °C		60 °C		
	1 m	3 m	14 d	1 m	7 d	21 d	7 d	14 d	1 d	7 d	
FM	√	√	√	√	√	√	√	√	√	√	5
M1	√	√	√	√	√	√	√	√	-	-	4
M2	√	√	√	√	√	√	-	-	√	√	4
M3	√	√	√	√	-	-	√	√	√	√	4
M4	√	√	-	-	√	√	√	√	√	√	4
M5	√	√	√	√	√	√	-	-	-	-	3
M6	√	√	√	√	-	-	√	√	-	-	3
M7	√	√	√	√	-	-	-	-	√	√	3
M8	√	√	-	-	√	√	√	√	-	-	3
M9	√	√	-	-	√	√	-	-	√	√	3
M10	√	√	-	-	-	-	√	√	√	√	3
M11	-	-	-	-	√	√	√	√	√	√	3
M12	√	√	-	-	√	√	-	-	-	-	2

√ included in ASAP evaluation, - not included in ASAP evaluation.

**Table 3:** Actual and predicted long-term stability data (5 ± 3 °C) for impurities of the full model (FM).

Impurity	Diol, %		Ethyl Ether, %		Total, %	
Time Point, Month	Actual	Predicted	Actual	Predicted	Actual	Predicted
0	not detected	not detected	not detected	not detected	0.11	0.11
6	0.06	0.04	0.04	0.05	0.29	0.32
12	0.09	0.09	0.11	0.10	0.54	0.54
24	0.17	0.18	0.16	0.20	1.06	0.96

**Table 4:** Actual and predicted long-term stability data (5 ± 3 °C) for impurities and statistical parameters of reduced four-temperature and full models.

				**6 m**		**12 m**		**24 m**	
		**R^2 a^**	**Q^2 b^**	**A, % ^c^**	**P, % ^d^**	**A, % ^c^**	**P, % ^d^**	**A, % ^c^**	**P, % ^d^**
FM	Diol	0.999	0.997	0.06	0.04	0.09	0.09	0.17	0.18
	Ethyl ether	0.998	0.995	0.04	0.05	0.11	0.10	0.16	0.20
	Total	0.997	0.991	0.29	0.32	0.54	0.54	1.06	0.96
M1	Diol	0.998	0.992	0.06	0.05	0.09	0.10	0.17	0.19
	Ethyl ether	0.998	0.990	0.04	0.06	0.11	0.11	0.16	0.22
	Total	0.995	0.973	0.29	0.34	0.54	0.57	1.06	1.03
M2	Diol	0.999	0.998	0.06	0.04	0.09	0.09	0.17	0.18
	Ethyl ether	0.999	0.997	0.04	0.05	0.11	0.10	0.16	0.20
	Total	0.998	0.994	0.29	0.32	0.54	0.52	1.06	0.93
M3	Diol	0.999	0.996	0.06	0.04	0.09	0.09	0.17	0.18
	Ethyl ether	0.998	0.994	0.04	0.05	0.11	0.10	0.16	0.21
	Total	0.997	0.989	0.29	0.32	0.54	0.53	1.06	0.96
M4	Diol	1.000	0.998	0.06	0.04	0.09	0.08	0.17	0.17
	Ethyl ether	0.999	0.998	0.04	0.05	0.11	0.09	0.16	0.19
	Total	0.999	0.997	0.29	0.30	0.54	0.49	1.06	0.86

^a^ Coefficient of determination (R^2^), ^b^ predictive relevance (Q^2^), ^c^ actual results (A), ^d^ predicted results (P).

**Table 5:** Actual and predicted long-term stability data (5 ± 3 °C) for impurities and statistical parameters of reduced three-temperature and full models.

				6 m		12 m		24 m	
		R^2 a^	Q^2 b^	A, % ^c^	P, % ^d^	A, % ^c^	P, % ^d^	A, % ^c^	P, % ^d^
FM	Diol	0.999	0.997	0.06	0.04	0.09	0.09	0.17	0.18
	Ethyl ether	0.998	0.995	0.04	0.05	0.11	0.10	0.16	0.20
	Total	0.997	0.991	0.29	0.32	0.54	0.54	1.06	0.96
M5	Diol	0.996	0.933	0.06	0.04	0.09	0.09	0.17	0.17
	Ethyl ether	0.994	0.895	0.04	0.05	0.11	0.10	0.16	0.20
	Total	0.990	0.827	0.29	0.31	0.54	0.50	1.06	0.88
M6	Diol	0.998	0.926	0.06	0.05	0.09	0.10	0.17	0.19
	Ethyl ether	0.997	0.897	0.04	0.06	0.11	0.11	0.16	0.22
	Total	0.995	0.784	0.29	0.34	0.54	0.57	1.06	1.04
M7	Diol	0.999	0.953	0.06	0.04	0.09	0.09	0.17	0.18
	Ethyl ether	0.999	0.935	0.04	0.05	0.11	0.10	0.16	0.20
	Total	0.998	0.860	0.29	0.32	0.54	0.52	1.06	0.93
M8	Diol	1.000	0.994	0.06	0.04	0.09	0.09	0.17	0.18
	Ethyl ether	1.000	0.996	0.04	0.05	0.11	0.10	0.16	0.20
	Total	0.998	0.975	0.29	0.31	0.54	0.52	1.06	0.92
M9	Diol	1.000	1.000	0.06	0.04	0.09	0.08	0.17	0.16
	Ethyl ether	1.000	1.000	0.04	0.05	0.11	0.09	0.16	0.18
	Total	1.000	0.999	0.29	0.30	0.54	0.48	1.06	0.85
M10	Diol	1.000	0.988	0.06	0.04	0.09	0.08	0.17	0.16
	Ethyl ether	0.999	0.985	0.04	0.05	0.11	0.09	0.16	0.19
	Total	0.999	0.976	0.29	0.29	0.54	0.48	1.06	0.84
M11	Diol	0.998	0.977	0.06	0.04	0.09	0.08	0.17	0.16
	Ethyl ether	0.998	0.976	0.04	0.04	0.11	0.09	0.16	0.17
	Total	0.996	0.940	0.29	0.31	0.54	0.50	1.06	0.88

^a^ Coefficient of determination (R^2^), ^b^ predictive relevance (Q^2^), ^c^ actual results (A), ^d^ predicted results (P).

**Table 6:** Actual and predicted long-term stability data (5 ± 3 °C) for impurities and statistical parameters of reduced model M12 and full model.

				12 m		24 m	
		R^2 a^	Q^2 b^	A, % ^c^	P, % ^d^	A, % ^c^	P, % ^d^
FM	Diol	0.999	0.997	0.09	0.09	0.17	0.18
	Ethyl ether	0.998	0.995	0.11	0.10	0.16	0.20
	Total	0.997	0.991	0.54	0.54	1.06	0.96
M12	Diol	1	-	0.09	0.08	0.17	0.15
	Ethyl ether	1	-	0.11	0.08	0.16	0.16
	Total	1	-	0.54	0.48	1.06	0.95

^a^ Coefficient of determination (R^2^), ^b^ predictive relevance (Q^2^), ^c^ actual results (A), ^d^ predicted results (P).

**Table 7:** Statistical parameters of different formulations (F) obtained with ASAP prediction of M11.

	F1			F2			F3		
Statistical Parameter	Diol, %	Ethyl Ether, %	Total, %	Diol, %	Ethyl Ether, %	Total, %	Diol, %	Ethyl Ether, %	Total, %
R^2 a^	1.000	1.000	0.994	1.000	1.000	0.998	1.000	1.000	1.000
Q^2 b^	1.000	0.996	0.917	1.000	0.997	0.974	0.999	0.995	0.994

^a^ Coefficient of determination (R^2^), ^b^ predictive relevance (Q^2^).

## Data Availability

Data are contained within the article and Supplementary Materials.

## Supplementary Materials

The following supporting information can be downloaded at: https://www.mdpi.com/article/10.3390/pharmaceutics17020160/s1, Table S1: Stability data for critical individual impurities and total impurities; Table S2: Calculated relative differences between actual and predicted stability data.
