# Supplementary material for: Accelerated Predictive Stability Testing: Accelerating Registration Phase and Application of Reduced Designs for Shelf-Life Determination of Parenteral Drug Product
Source: Pharmaceutics. 2025 Jan 25;17(2):160. doi: 10.3390/pharmaceutics17020160 (PMC11858995; doi:10.3390/pharmaceutics17020160)
Supplement: Supplementary file 1 [file pharmaceutics-17-00160-s001.zip › pharmaceutics-3429011-supplementary.pdf]

pharmaceutics

MDPI

Supplementary Materials: Accelerated Predictive Stability Testing: Accelerating Registration Phase and Application of Reduced Designs for Shelf-Life Determination of Parenteral Drug Product

Lara Pavčnik, Mateja Prunk, Tina Trdan Lušin and Robert Roškar

Table S1. Stability data for critical individual impurities and total impurities.

Formulation	Stability condition	Time point, months (m), days (d)	Diol, %	Ethyl ether, %	Total, %
Prototype	Initial	0	not detected	not detected	0.113
	5 °C ± 3 °C	3 m	0.035	0.053	0.222
	5 °C ± 3 °C	6 m	0.056	0.041	0.286
	5 °C ± 3 °C	12 m	0.088	0.106	0.544
	5 °C ± 3 °C	24 m	0.169	0.164	1.063
	25 °C ± 2 °C/60% RH ± 5% RH	1 m	0.096	0.130	0.485
	25 °C ± 2 °C/60% RH ± 5% RH	3 m	0.254	0.304	1.385
	25 °C ± 2 °C/60% RH ± 5% RH	6 m	0.496	0.572	3.094
	30 °C ± 2 °C/65% RH ± 5% RH	14 d	0.073	0.109	0.356
	30 °C ± 2 °C/65% RH ± 5% RH	1 m	0.170	0.213	1.059
	40 °C ± 2 °C/75% RH ± 5% RH	7 d	0.108	0.154	0.488
	40 °C ± 2 °C/75% RH ± 5% RH	21 d	0.326	0.406	1.952
	50 °C ± 2 °C/75% RH ± 5% RH	7 d	0.292	0.391	1.546
	50 °C ± 2 °C/75% RH ± 5% RH	14 d	0.557	0.728	3.222
	60 °C ± 2 °C/75% RH ± 5% RH	1 d	0.133	0.193	0.682
	60 °C ± 2 °C/75% RH ± 5% RH	7 d	0.775	1.025	4.878
Formulation 1	Initial	0	not detected	not detected	0.115
	40 °C ± 2 °C/75% RH ± 5% RH	7 d	0.091	0.101	0.387
	40 °C ± 2 °C/75% RH ± 5% RH	21 d	0.266	0.308	1.019
	50 °C ± 2 °C/75% RH ± 5% RH	7 d	0.261	0.318	1.138
	50 °C ± 2 °C/75% RH ± 5% RH	14 d	0.537	0.680	2.982
	60 °C ± 2 °C/75% RH ± 5% RH	1 d	0.107	0.128	0.464
	60 °C ± 2 °C/75% RH ± 5% RH	6 d	0.646	0.829	3.664
Formulation 2	Initial	0	not detected	not detected	0.114
	40 °C ± 2 °C/75% RH ± 5% RH	7 d	0.097	0.109	0.397
	40 °C ± 2 °C/75% RH ± 5% RH	21 d	0.283	0.337	1.215
	50 °C ± 2 °C/75% RH ± 5% RH	7 d	0.272	0.338	1.167
	50 °C ± 2 °C/75% RH ± 5% RH	14 d	0.571	0.729	3.105
	60 °C ± 2 °C/75% RH ± 5% RH	1 d	0.112	0.138	0.473
	60 °C ± 2 °C/75% RH ± 5% RH	6 d	0.676	0.876	3.859
Formulation 3	Initial	0	not detected	not detected	0.114
	40 °C ± 2 °C/75% RH ± 5% RH	7 d	0.102	0.122	0.416
	40 °C ± 2 °C/75% RH ± 5% RH	21 d	0.309	0.382	1.543
	50 °C ± 2 °C/75% RH ± 5% RH	7 d	0.298	0.385	1.356

 https://doi.org/10.3390/xxxxx

50 °C ± 2 °C/75% RH ± 5% RH	14 d	0.624	0.823	3.556
60 °C ± 2 °C/75% RH ± 5% RH	1 d	0.124	0.154	0.494
60 °C ± 2 °C/75% RH ± 5% RH	6 d	0.733	0.976	4.350

Table S2. Calculated relative differences between actual and predicted stability data.

**Model**	No. of temperatures	12 months			24 months		
		Diol, %	Ethyl ether, %	Total, %	Diol, %	Ethyl ether, %	Total, %
FM	5	0	10	0	6	22	10
M1	4	11	0	5	11	32	3
M2		0	10	4	6	22	13
M3		0	10	2	6	27	10
M4		12	20	10	0	17	21
M5	3	0	10	8	0	22	19
M6		11	0	5	11	32	2
M7		0	10	4	6	22	13
M8		0	10	4	6	22	14
M9		12	20	12	6	12	22
M10		12	20	12	6	17	23
M11	3	12	20	8	6	6	19
M12	2	12	32	12	13	0	11
